# Experiences of Indigenous and ethnic minority women with culturally safe healthcare in Europe: A scoping review

**DOI:** 10.1371/journal.pone.0325847

**Published:** 2025-06-25

**Authors:** Garbiñe Elizegi Narbarte, Iratxe Perez-Urdiales, Jennifer James, Leslie Dubbin, Stella Bialous Aguinaga

**Affiliations:** 1 Department of Social and Behavioral Sciences, University of California San Francisco, San Francisco, California, United States of America; 2 Department Nursing I, University of the Basque Country (UPV/EHU), Leioa, Bizkaia, Basque Country; University of Greenwich, UNITED KINGDOM OF GREAT BRITAIN AND NORTHERN IRELAND

## Abstract

Worldwide, Indigenous and ethnic minority women encounter discrimination in access to high quality healthcare and other advantageous social determinants of health. Cultural safety is the concept of proactively considering social, economic, and political situations, and power relationships in healthcare. By identifying ways in which culturally unsafe healthcare practices can intensify institutional discrimination and replicate traumatic experiences in historically oppressed populations, interventions may be crafted to improve patient experiences and outcomes. The purpose of this paper is to conduct a scoping review of research on experiences with culturally safe healthcare among adult Indigenous and ethnic minority women in Europe. All research articles within Europe, without set date parameters, addressing the experiences of individuals who self-identify as adult women or gender non-normative individuals who are members of Indigenous or ethnic minority communities were included. A total of four peer reviewed articles were identified for this scoping review. Participants in four studies described healthcare providers’ lack of knowledge of their culture and healthcare needs. The studies suggest that this lack of knowledge may lead to patient sentiments of inferiority, prejudice, increased barriers to access care, inadequate healthcare intervention and ineffective healthcare service. The articles propose the implementation of cultural safety to close the gap of health disparities in Indigenous and ethnic minority populations. There are limited data on the implementation of cultural safety in Europe, potentially indicating a lack of awareness regarding the concept of cultural safety or its core tenets, as well as regarding the importance of culture, racism and biases in healthcare related to ethnic minority populations. Overall, this scoping review reiterates the gap in research and knowledge in the implementation of culturally safe healthcare in Europe.

## Introduction

Worldwide, Indigenous and ethnic minority women encounter disparities in their exposure to social determinants of health, as well as access to high quality healthcare [1,2]. While these distinct groups are highly diverse (i.e., language, culture, migration status, origin, etc.), they share common challenges such as higher levels of poverty, lack of access to social services, gaps in social protection coverage and higher inequalities in healthcare outcomes [3]*.* Europe is home to over 100 ethnic minority groups [4], which form many of Europe’s “stateless nations” [5], and are distinguished from other minorities in their claim for self-determination [6]. The Basque Country, which is administratively divided in two nation-states (Spain and France) [7] or Sápmi which is divided in four nation-states (Finland, Norway, Sweden and Russia) [8] are examples of many stateless nations in Europe. Both the Basque and Sámi people have endured centuries of systemic oppression, marked by forced assimilation policies. These measures included taxation, legal restrictions, and religious regulations, alongside efforts to suppress their languages through the education system [9,10].

In the Basque Country, the healthcare system continues to reflect the historical imposition on the Basque language, with a shortage of bilingual professionals who speak Basque [11,12]. The Basque population’s legal right to access healthcare services in their language depends on the varying legal status of the Basque language across different administrative regions within the Basque Country. As a result, patients often struggle to communicate in their preferred language [13], which can hinder their ability to fully understand medical options and make informed healthcare decisions.

The Sámi people are entitled to healthcare services under both national and international legal frameworks. In Norway, their rights are protected by general legislation, such as the Patients’ Rights Act, and international agreements like the UN Declaration on the Rights of Indigenous Peoples and the International Covenant on Civil and Political Rights, which have been incorporated into Norwegian law through the Human Rights Act [9]. Despite these legal protections, research suggests that the Sámi report lower satisfaction with healthcare services compared to the majority population [14,15]. However, their health challenges differ from those faced by Indigenous groups in Canada, the United States, Russia, and Greenland. In Norway, Sámi and non-Sámi individuals have comparable life expectancies and similar mortality rates for specific health conditions [9]. Nevertheless, certain groups within the Sámi population, such as reindeer herders, appear to experience higher levels of depression and anxiety than the majority population in the same regions [16].

Research indicates that while all Indigenous, and ethnic minority groups experience communication/language problems, cultural and religious differences, and discrimination during healthcare encounters this problem is even more pronounced in women within these groups [17–19]*.* A woman´s origin, culture, geographic background, and access to healthcare can impact their experiences [20]. Consequently, minority women have a unique perspective of women´s healthcare issues that are not adopted by other groups, which can create isolation and could function as an impediment to social action or health behaviors considered acceptable by the majority of women [21].

Many minority language speakers and non-hegemonic cultures are denied access to quality healthcare services due to the power differentials that exist between the minority and dominant cultures. According to Roche [22], language oppression, as a form of domination, is comparable to oppression related to race, color, national origin, or ethnicity. While many European countries have multiple official languages and more than 50% of Europeans can communicate in more than one language—exposing them to diverse cultures and worldviews—the continent is also home to numerous regional and ancestral languages. However, studies on the impact of culture and language in healthcare remain scarce [23]. Aside from studies performed on the Catalans, Sámi, and Swedish community in Norway that address healthcare service issues in bilingual settings, other European studies regarding language and healthcare have mainly focused on immigrant populations and lack of standardized tools for other groups [23].

The concept of cultural safety has been promoted by Mãori nurses working in Aotearoa within a colonial context, and other Indigenous, and ethnic minority communities in Australia and Canada [24]. Few studies regarding cultural safety have been conducted in Europe [23]. According to Curtis et al. [1], in addition to the power differential arising from language and culture, other contributing factors include an overall lack of awareness and training of healthcare workers regarding the importance of culture and language associated with a particular group within the healthcare delivery system. In these situations, therapeutic relationships between a healthcare worker and a patient are particularly at risk of intended or unintended bias. For safe and quality care to be implemented, healthcare workers need to work towards both cultural safety and critical consciousness. The role of health workers and healthcare systems in generating and sustaining these inequities is progressively undergoing scrutiny [1].

Cultural safety is the concept of proactively and consciously considering social, economic and political situations, and power relations in healthcare, acknowledging that culturally unsafe healthcare practices can intensify institutional discrimination and replicate traumatic experiences in historically oppressed populations [25]. Cultural safety addresses the power imbalances between practitioners and patients through the process of reflexivity. That is, health care providers, and health systems, must acknowledge and address their own cultural biases and recognize that each individual’s dignity must be valued. Cultural safety is a concept that derives from critical social theory and asserts that established methods such as, ‘cultural awareness’ or ‘cultural competence’, which are often applied in healthcare, neglect to address power relationships, which are historically unbalanced between migrant and/or Indigenous, and ethnic minority groups and healthcare providers and services [26]. As such, Indigenous, and ethnic minority groups are often perceived as the ‘other,’ rather than as an ally whose knowledge and values can positively contribute to the patient-provider relationship or improve healthcare services. Culturally safe practice, on the other hand, focuses on relationships of both mutual and reciprocal trust and respect [26]. Healthcare organizations and authorities must also be held accountable for delivering culturally safe health care that meets the needs of individuals and their communities [1].

Despite global attempts to increase patient safety and care quality, improving safety for Indigenous and ethnic minority populations has lacked sufficient attention and thus, remains an under researched area. As argued by Chauhan [27], patients from Indigenous, and ethnic minority groups continue to feel unsafe, experience discrimination, lack appropriate interpreting services and have inadequate knowledge of the healthcare settings/systems during healthcare encounters [27]. Therefore, the purpose of this scoping review is to analyze and summarize the available literature related to experiences with culturally safe care among Indigenous and ethnic minority women (IEMW) in Europe.

## Materials and methods

### Protocol and registration

Scoping reviews represent a methodological approach to knowledge synthesis that systematically maps the existing literature on a defined topic, with the aim of identifying key concepts, theoretical frameworks, sources of evidence, and research gaps [28,29]. This scoping review will follow the Preferred Reporting Items for Systematic Reviews and Meta-Analyses (PRISMA) Extension for Scoping Reviews (PRISMA-ScR), which includes a 27 item checklist (See Appendix A) [28].

### Eligibility criteria

This review included studies involving IEMW from all European countries and stateless nations. Studies addressing the health care experiences of IEMW who self-identify as Indigenous or as members of an ethnic minority group have been included. Excluded were studies where the participant was not the recipient of the healthcare service, or if the study focused only on the experience of the healthcare professional [30]. No publication date restrictions were applied. Inclusion and exclusion criteria are shown in Table 1.

**Table 1:** Scoping review inclusion and exclusion criteria.

	Inclusion criteria	Exclusion criteria
**Sample**	Adult Indigenous/ethnic minority women and gender non-normative individuals exposed to culturally safe healthcare in Europe.	Under 18 years old. Studies of Indigenous/ethnic minority women and gender non-normative individuals outside of Europe.
**Phenomenon of Interest**	Indigenous, and ethnic minority women and gender non-normative individuals´ experiences with culturally safe healthcare in Europe.	Studies examining experiences of healthcare providers only. Studies that do not address the Indigenous or ethnic or do not include women
**Design**	Literature review and research articles	Protocols, opinion pieces, anecdotal reports, editorials, news articles, and dissertations.
**Evaluation**	Patient’s experiences with quality of care, language, and cultural safety during a healthcare encounter.	Studies focused only on social determinants of health which do not address the historical content and power relations within the healthcare encounter. Studies focused on patient safety regarding adverse events, medication errors, diagnostic errors.
**Research Type**	All research studies, meta-analysis, meta-synthesis, systematic reviews, and scoping reviews	Non-databased papers
**Report Characteristics**	Full texts available in all languages with abstracts in English No publication date restrictions	Protocols, opinion pieces, anecdotal reports, editorials, news articles, dissertations

For this study we defined culturally safe care as safe, quality care as defined by the participant and/or their community [30]. Studies that did not focus on core tenets of cultural safety—such as community relationships, cultural identity, and power relations [30] or that did not use a cultural safety framework or explicitly engage with its core principles were excluded. Due to the diverse use and overall lack of consensus with regards to the concepts of cultural safety and related terms, studies which also address concepts such as cultural competency, cultural humility have been considered if previously defined inclusion criteria was met [1].

### Information sources

The five databases searched for relevant studies were PubMed, CINAHL (EBSCOhost), Embase, Web of Science, and Epistemonikos.

#### Search.

A professional librarian assisted in the development of a systematic search strategy utilizing a combination of Medical Subject Headings (MeSH) and related terms to identify studies on experiences of Indigenous, and ethnic minority women and gender non-normative individuals with culturally safe care within the European territory (See Appendices B, C, D, E, and F). Only full text research articles were included. Protocols, opinion pieces, anecdotal reports, editorials, news articles, and dissertations were excluded from the search (Table 1). The initial search was conducted between March 15 and May 15 of 2023 and was updated in January of 2025 [31].

#### Selection of sources of evidence.

All identified articles were imported into Zotero [32] citation software to perform an initial removal of duplicate articles. The articles were subsequently imported to Covidence software [33] to prepare for title and abstract review and to create data charts. The articles selected for screening were examined by the lead researcher (G.E.) and a secondary reviewer (S.B.) who independently screened the articles by title and abstract, following the established eligibility criteria [31]. Full text articles that were assessed as possibly meeting eligibility criteria on the initial screening were then reviewed to evaluate full eligibility criteria.

#### Data charting process.

Data extraction and charting was conducted by utilizing an adapted Covidence software application incorporating items defined in the Joanna Briggs Institute (JBI) Manual for Evidence Synthesis of Scoping Reviews [34]. Following JBI guidelines, data items were incorporated and revised as necessary in an iterative process to guarantee inclusion of relevant data.

#### Data extracted.

Data extracted included the following study characteristics: country of origin; year; aims; participants; setting; sampling criteria; age; and gender. In addition, the methodology used (i.e., data collection and analysis), and key findings/outcomes were also extracted.

None of the selected articles included the experiences of gender non-normative individuals who were also members of Indigenous or ethnic minority communities. Due to the limited number of articles exclusively focusing on the experiences of Indigenous and ethnic minority women, all studies meeting the above criteria were included if there were any women participants included, i.e., as part of the sample even if not exclusively.

#### Critical appraisal of individual sources of evidence.

This scoping review did not include a quality appraisal. Scoping reviews are intended to supply an overview, or “map” evidence relative to the phenomenon of interest, rather than critically appraising and synthesizing data related to a specific question. Therefore, quality appraisals are generally not included in scoping reviews [35].

### Synthesis of results

To provide a broad overview of the research topic, all results pertaining to the studied concepts and experiences that were reported in the literature were also reported in this review. Each study´s sample, aims, methodology, outcomes, and key findings were summarized and analyzed by both reviewers.

## Results

### Selection of sources of evidence

**Fig 1:**
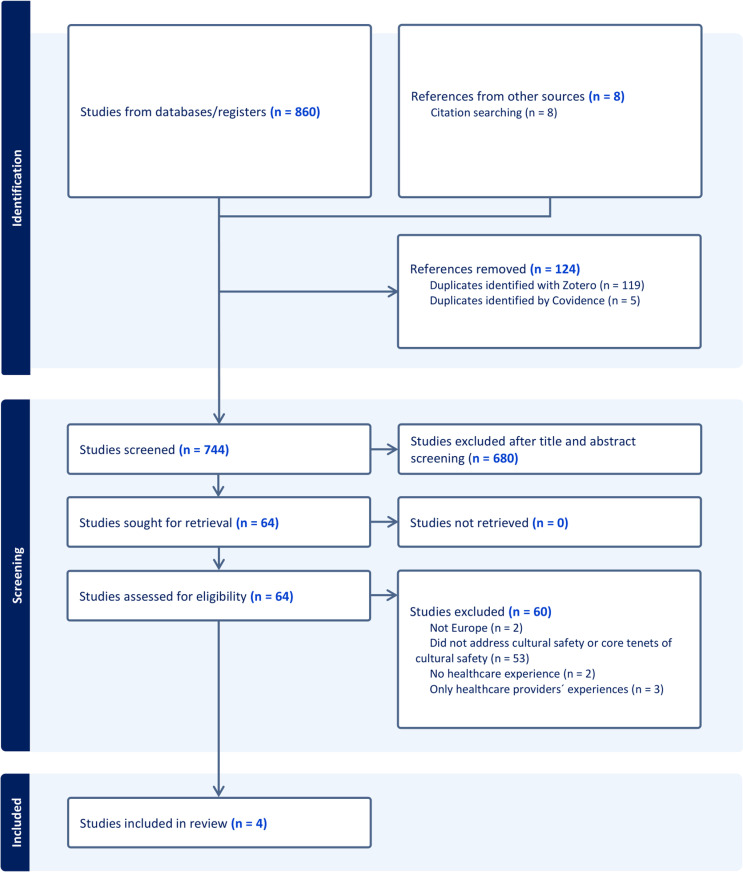
Flow diagram of evidence search.

Following the scoping review framework outlined above, multiple databases and reference lists were searched to produce a broad list of relevant studies (Fig 1). The primary search resulted in a total of 860 articles found in the following databases: PubMed (n = 227); CINAHL (n = 89); Embase (n = 28); Web of Science (n = 521) and Epistemonikos (n = 0). An additional 8 articles were retrieved from additional sources, such as reference lists and broad web-based searches conducted prior to this review, increasing the total to 868 articles. Subsequently, 124 duplicates were identified by using the Zotero and Covidence software. Therefore, the remaining 744 articles were scanned to review the title and abstract for possible inclusion. Title and abstract screening resulted in the removal of 680 (91%) articles, which produced the retrieval of 64 (9%) articles. After reading in full the 64 remaining articles, additional exclusions were conducted for studies that did not address cultural safety or core tenets thereof, such as relationship of community, cultural identity and power relations (n = 53; 83%), studies that were not conducted in Europe (n = 2; 3%), did not include healthcare experiences of participants (n = 2; 3%), or only addressed the experiences of healthcare providers (n = 3; 5%). Ultimately, 4 articles were retained for this scoping review.

### Characteristics of sources of evidence

All four studies were published in English in peer-reviewed international open access journals. Two were articles from a journal that specializes in circumpolar health and has a specific interest in Indigenous peoples [14,15]. One article was published in a journal focused on qualitative research studies related to health and well-being [36], and one was published in a journal that publishes a wide variety of research studies that cover all aspects of medicine and healthcare [37].

### Samples

Sample size of participants varied from eleven [14] to forty eight [36] (Table 2). Two articles [14,15] include Sámi participants in Norway. Olaniyan and Hayes [36] incorporated 48 Black British (n = 32) and South Asian-British (n = 16) students from racial and ethnic minority backgrounds living in the United Kingdom, who were attending a higher education institution. Thirteen participants had a current professional diagnosis of depression (n = 8), anxiety (n = 4) or bipolar disorder (n = 1), 21 had self-defined mental health problems and 25 had prior help-seeking experiences. Del Pino, et al. [37] included 16 adult Romani women (RW) aged between 23 and 62 who had contact with the public health system. Samples were obtained through purposeful, convenience and snowball sampling.

**Table 2:** Characteristics of sources of evidence.

Study ID	Origin/Country	Title	Setting	Participants
Mehus et al. [14]	Hammerfest, Norway	Exploring why and how encounters with the Norwegian health-care system can be considered culturally unsafe by North Sámi-speaking patients and relatives: A qualitative study	Two inland Sámi administrative municipalities in northern Norway.	North Sámi participants (n = 11), self-identified Sámi-speaking and Sámi. Women (n = 9), Men (n = 2). Mean age (47.5) years, 56.5 for men and 45.5 for women. Former patients (n = 4) or relatives to a patient (n = 7).
del Pino et al. [37]	Almeria (Andalusia-Spain)	Romani Women and Health: The Need for a Cultural-Safety Based Approach	Peripheral area of the city of Almeria (Spain), in an area of medium-low socio-economic level with slums and deficient city maintenance and hygiene.	16 Romani women who had contact with the Public Health System. Medium-low socio-economic status. Mean age (38.4) mean # of children (4.8). 12 participants married, one single, and one widowed.
Ness and Munkejord [15]	Norway	“All I expect is that they accept that I am a Sámi” an analysis of experiences of healthcare encounters and expectations for future care services among older South Sámi in Norway	Participants from scattered communities in rural parts of Mid- and Northern Norway.	Older, self-identified, South Sámi (n = 12), women (n = 5), men (n = 7). Aged between 67 and 84 years (mean age n = 74). All had background from reindeer herding families. All had experience with the healthcare system as a care receiver.
Olaniyan and Hayes [36]		Just ethnic matching? Racial and ethnic minority students and culturally appropriate mental health provision at British universities	Two neighboring British Higher Education Institutions. Both institutions are selective and high performing.	UK domiciled racial and ethnic minority students (n = 48). Women (n = 30; 62.5%) Men (n = 18; 37.5%). Black British (n = 32) and South Asian-British background (n = 16). 13 participants with current professional diagnosis of mental illness, 21 with self-defined mental health problems and 25 with prior help-seeking experiences.

### Design and methodology

Qualitative methodologies were used in all four studies (Table 3). A constructivist grounded theory (CGT) approach was used in two studies [15,36], and interpretive phenomenology (IP) was used in another study [37]. Mehus et al. [14] conducted a qualitative explorative descriptive study. Semi-structured qualitative interviews were conducted in all four studies followed by data coding and thematic analysis.

**Table 3:** Aims, methods, and key findings of sources of evidence.

Study ID	Aims	Methodology/Methods	Outcomes	Key Findings
Mehus et al. [14]	To explore how Sámi-speaking patients and relatives experience healthcare encounters	Methodology: Qualitative explorative, descriptive study. Data collection: Semi-structured interviews were conducted. Data was collected in two inland Sámi administrative municipalities in northern Norway from April to October 2015. Data analysis: Content analysis was performed using a conceptual lens of cultural safety.	Data analysis resulted in two outcome groups: 1) Contributions to the feeling of being culturally safe 1.1 Meeting Sámi-speaking staff and patients 1.2 Having Sámi activities and symbols in hospitals. 1.3 Meeting staff that listen and spend time with patients. 1.4 Having interpreting services. 1.5 Having the feeling of being home. 1.6 Meeting other Sámi-speaking patients 2) Contributions to the feeling of being culturally unsafe 2.1 Not using Sámi language and not being allowed to speak Sámi in public. 2.2 Feeling violated, invisible and vulnerable without Sámi music, art and handcraft. 2.3 Staff talking above your head and giving no information. 2.4 Neglect of Sámi language and no offer of interpreting service 2.5 Not feeling at home. 2.6 No one to speak Sámi with	-All participants described suboptimal experiences of what was identified as cultural safety in healthcare due to limited use of Sámi language or professional interpreters, and perceived discrimination. -This experience was perceived at an institutional, group, and individual level. -There were limited Sámi-speaking staff, staff did not reflect on the importance of interpreting for patient safety, there is no cultural validation of medical tests, interpreters were not prepared for healthcare encounters, and Sámi art and music were less present in hospitals outside Sámi areas. -Highlighting the concept of culturally safe care as a goal at individual, group and institutional level was recommended. -This includes addressing power imbalances and inequitable social relationships in healthcare.
del Pino et al. [37]	To delve into the perceptions of Romani Women on their health beliefs and experiences with health services and their health professionals as well as the role of Romani Women in the health of their community.	Methodology: Qualitative study using an interpretative phenomenological approach. Data collection: Semi-structured interviews were conducted in October 2021. Data Analysis: Data was analyzed through coding and thematic analysis.	Themes: Romani Identity, Social and Economic Conditions, Health and Sickness Concepts, Experiences with Health Services. Categories: Construction of the Romani Woman Identity, Difficulties in Life, Health and Sickness Beliefs, Barriers Health System Access	-RW played a significant role in their community, which made them essential participants in any collective health promotion program. -Romani cultural elements were not recognized by health professionals creating conflict in the healthcare setting. -Lack of knowledge of healthcare professionals regarding Romani beliefs resulted in inadequate health intervention and ineffective healthcare service. -Prejudice and stereotyping were present during the healthcare encounter and it increased the barriers to healthcare access for Romani Women. -Incorporation of intercultural mediators and Romani health professionals was proposed to close build bridges between Romani and non-Romani people. -Addressing the social determinants of health, difficulty of access to healthcare services, prejudice, and cultural differences was recommended to improve healthcare outcomes for the Romani collective. -Health interventions with the Romani community must consider the principles of Cultural Safety.
Ness and Munkejord [15]	1) To forward empirical knowledge of how older Sámi experience healthcare encounters in Norway and what they expect in terms of future care services 2) To forward understanding of how more culturally safe services could be offered to the Sámi population	Methodology: Qualitative, interpretative and constructivist grounded theory research design. Data collection: Semi-structured in-depth interviews were conducted. Data analysis: Reflexive thematic approach was used to analyze the data.	Themes: 1) Ambivalent healthcare encounters 1.1 “Sometimes we feel deprioritized.” 1.2 “Sometimes we just don’t understand each other” 1.3 “They don’t know anything about us.” 2) Future expectations about healthcare services in the municipality 2.1 “I hope they will accept me”	-Healthcare encounters were experienced as culturally ambivalent by South Sámi care receivers. -Participants felt deprioritized and misunderstood in healthcare encounters. -Healthcare professionals did not have enough time for the participants -When participants encountered a “nice” general practitioner they tried to see this specific practitioner. -Healthcare professionals did not know anything about Sámi culture and their history, this occasionally led to ignorance and prejudice. -This lack of knowledge could have led to a feeling of inferiority amongst Sámi patients. -“Standardized services” may contribute to masking discrimination. -Hopes for the future of participants were that they would be “accepted” and “respected” as Sámi.
Olaniyan and Hayes [36]	To explore how racial and ethnic minority students define culturally appropriate support and the approaches they view to be effective in promoting help-seeking	Methodology: Qualitative study using constructive grounded theory principles. Data collection: Semi-structured interviews were conducted between December 2018 and November 2019; 22 in-person interviews, and 26 phone interviews. Data analysis: Data analysis was guided by principles of constructivist grounded theory and reflexive thematic analysis.	1) Participants consistently recognize the lack of diversity to be a barrier to mental health help-seeking on campus. 2) Although for some participants ethnic matching was crucial, they would rarely advocate for this to be the only solution to the problem. 3) For British South Asian participants ethnic matching would be harmful. 4) Ethnic matching was also viewed as a “bailout” for White mental health practitioners and keeping them from engaging in racial differences. 5) This would also leave the responsibility of solving health inequalities within the affected individuals and communities. 6)Practitioners should engage in reflexivity and address the potential impact of their own culture in their practice 7) Beyond their background, participants desire a “person specific” approach	-The findings demonstrated the need for increased sensitivity in the way mental health support is provided in higher education institutions. -Findings demonstrated more than one recommended way to provide culturally appropriate care. -Majority of participants perceived ethnic matching or sharing a common background with providers beneficial. However, it is considered insufficient unless it is based on structural reform. -Campus mental health services were structured from a white Eurocentric perspective which was perceived as an accessibility barrier for REM students.

### Synthesis of evidence

### Results of individual sources of evidence

Outcomes and key findings of interest are summarized in Table 3, which briefly identify the aims, methodology, outcomes, and key findings. To contextualize findings, main outcome themes were also included in the synthesis.

Three studies specifically utilized cultural safety as a framework to address power relations between healthcare professionals and patients situated in a historical context of racism and discrimination which results in sub-optimal healthcare delivery [14,15,37]. Olaniyan and Hayes [36] refer to the need for “culturally appropriate” assistance for racial and ethnic minority students. However, the definition of culturally appropriate care proposed in the study by the authors and participants include concepts such as reflexivity, structural racism, power relations and cultural acknowledgment which are also key tenets of cultural safety [14].

Participants in all four studies describe a lack of knowledge of healthcare providers regarding their culture and consequently their healthcare needs. This lack of knowledge may lead to sentiments of inferiority, prejudice, [15] increased barriers to access care, inadequate healthcare intervention and ineffective healthcare service [37]. In one study, participants refer to the lack of diversity in the university’s support service staff to be a barrier to mental health help-seeking on campus [36]. Although for some participants ethnic matching or sharing an ethnic and cultural background with providers was crucial, they would rarely advocate for this to be the only solution to the problem. For British South Asian participants ethnic matching would potentially generate conflict and judgement from their extended community if the provider was also a member of this community. Ethnic matching was also viewed as a “bailout” for White medical health professionals and keeping them from engaging in racial differences [36].

Key findings in the evaluated studies included the proposal of various measures to implement culturally safe healthcare with the affected populations. Regarding ways to overcome cultural discordance, in the case of Romani women, incorporation of intercultural mediators and Romani health professionals were proposed to build bridges between Romani and non-Romani people. For Sámi participants “standardized services” within the Norwegian system may contribute to masking discrimination and hopes for the future of participants are that they would be “accepted” and “respected” as Sámi [15].

To overcome health system’s organizational barriers the social determinants of health, difficulty of access to healthcare services, prejudice, and cultural differences must be addressed to improve healthcare outcomes for the Romani collective and health interventions with the Romani community must consider the principles of cultural safety [37]. For racial and ethnic minority participants the findings demonstrate the need for increased sensitivity in the way mental health support is provided in higher education institutions, and participants advocate for a “person specific” approach that allows various ways to provide culturally appropriate care [36]. Highlighting the concept of culturally safe care as a goal at individual, group and institutional level is recommended including addressing power imbalances and inequitable social relationships in healthcare [14].

## Discussion

This review identified 4 sources related to experiences with culturally safe care among adult IEMW in Europe. The limited number of articles included highlights a significant gap in research utilizing cultural safety as a framework to address the experiences of Indigenous and ethnic minority individuals within Europe. All articles were peer-reviewed and published in English in international journals. Although all of the articles included women as participants, only one article was exclusively exploring the experiences of ethnic minority women [37], and none of the articles addressed the experiences of gender non-normative individuals.

In terms of sampling, all articles were published within the last 6 years, indicating the innovative standing of the implementation of cultural safety in health care. It also detects the scarcity of research addressing cultural safety within the European context. The samples varied in size, demographic characteristics of participants, and cultural backgrounds. This variety supports previous research encouraging the implementation of cultural safety interculturally [38].

Most articles in this review emphasized the necessity for implementation of cultural safety to close the gap of health disparities in Indigenous and ethnic minority populations. Participants in the reviewed studies referred feeling diminished, demeaned, and disempowered, which are considered the 3D´s of culturally unsafe practice [39]. Proposed measures to implement culturally safe healthcare in the studied settings included addressing the health disparities, power relations, and cultural aspects within the healthcare system. These findings coincide with research highlighting the importance of emphasizing the differences between healthcare workers and patients that influence care, and work to reduce any aggression on the patient’s cultural identity as a way to implement culturally safe healthcare [1].

Most of the proposed measures focused on healthcare provider training or reflexivity. However, few measures addressed the institutional responsibility for the implementation of the proposed measures and policies. This conflicts, to some degree, with studies that highlight the need to embed cultural safety at both individual and institutional levels [14,40], to avoid the risk of ignoring contexts by avoiding to draw from critical theoretical perspectives [38].

Most findings present ethnic matching or cultural and language concordance as an important aspect to improve cultural safety during the healthcare encounter. However, reviewed studies address that this is not as beneficial if cultural and historical contexts are disengaged. This matches research highlighting the significance of maintaining focus on providing a safe environment for Indigenous and ethnic minority patients, as defined by the community, during the healthcare encounter, which is one of the essential origins of cultural safety [41].

Overall, the reviewed studies acknowledge the lack of culturally safe care in distinct Indigenous and ethnic minority communities within Europe. There is also agreement in the lack of consensus with the use of cultural safety and related terms such as cultural competence, cultural awareness, cultural sensitivity, and culturally appropriate care [1,36], as well as risks of oversimplifying the concept by depriving it from critical theoretical perspective [40]. Despite the limited research on culturally safe care in the European context and the need to advance its implementation, universities in Norway have been engaged in an ongoing initiative since 2019 focused on developing learning outcomes to better meet the needs of Sámi patients. In addition, a Sámi nursing education program has been established to enhance culturally safe healthcare services [42]. The implementation of these programs could serve as valuable models for integrating culturally safe practices in healthcare settings serving other Indigenous and ethnic minority populations across Europe.

## Limitations

Although several databases were systematically searched, it is possible that data from unpublished studies, such as dissertations, were inadvertently overlooked in this scoping review. Our primary objective was to examine the use of cultural safety as a framework within the European context, and the strict inclusion criteria we applied consequently limited the number of articles included. This finding highlights that there is significant room for growth in this area. Furthermore, the lack of studies specifically focusing on Indigenous, Ethnic, and Migrant Women (IEMW) further constrained our ability to explore gender disparities and inequalities in relation to culturally safe healthcare.

## Conclusions

This scoping review shows that Indigenous and ethnic minority populations in Europe experience discrimination and receive suboptimal healthcare services. This appears to be related to power relations within the healthcare encounter and the healthcare system. There are limited data on the implementation of cultural safety in Europe, potentially indicating a lack of awareness regarding the concept of cultural safety or its core tenets, as well as regarding the importance of culture, racism and biases in healthcare related to ethnic minority populations. Overall, this scoping review reiterates the necessity for further studies to promote the implementation of cultural safety in Indigenous and ethnic minority in the European context.

## Supporting information

S1 Appendix A Prisma Checklist. (DOCX)

S2 Appendix B Search strategy for Database B. (DOCX)

S3 Appendix C Search strategy for Database C. (DOCX)

S4 Appendix D Search strategy for Database D. (DOCX)

S5 Appendix E Search strategy for Database E. (DOCX)

S6 Appendix F Search strategy for Database F. (DOCX)
