# Supplementary figures and images for: Experiences of Indigenous and ethnic minority women with culturally safe healthcare in Europe: A scoping review
Source: PLoS One. 2025 Jun 25;20(6):e0325847. doi: 10.1371/journal.pone.0325847 (PMC12193589; doi:10.1371/journal.pone.0325847)

**Appendix A: Prisma Checklist**


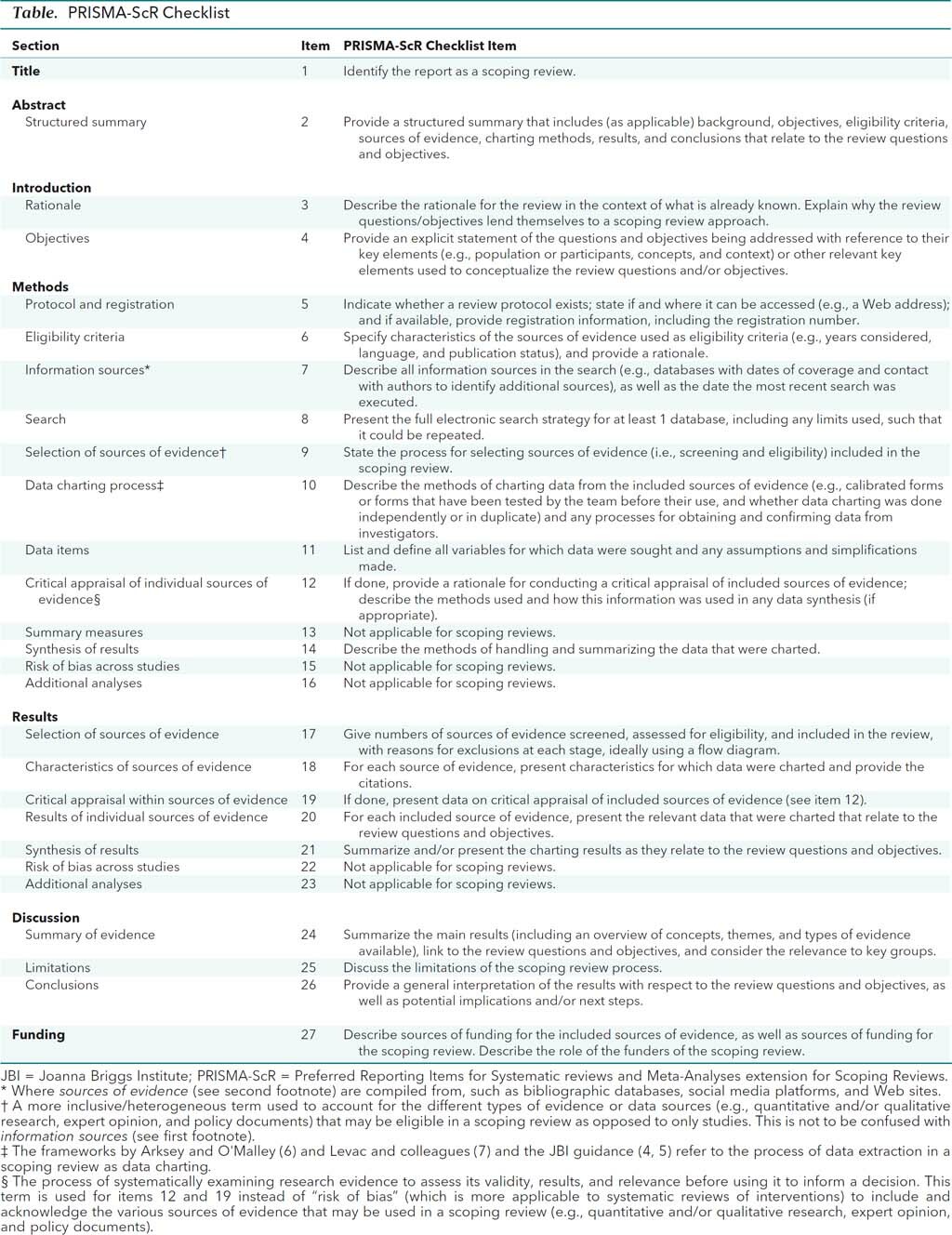

Supplement: S1 Appendix A — (DOCX) [file pone.0325847.s001.docx]
