# Supplementary material for: Experiences of Indigenous and ethnic minority women with culturally safe healthcare in Europe: A scoping review
Source: PLoS One. 2025 Jun 25;20(6):e0325847. doi: 10.1371/journal.pone.0325847 (PMC12193589; doi:10.1371/journal.pone.0325847)
Supplement: S3 Appendix C — (DOCX) [file pone.0325847.s003.docx]

**Appendix C: Search strategy by database**

| **#** | **CINAHL. The searches were completed on January 23, 2025. No date limits were applied to the searches. Title/Abstract** | |
| --- | --- | --- |
|  | **Search Syntax** | **# of Articles** |
| **1** | **(**Culturally safe care OR cultural safety OR cultural awareness OR culturally conscious OR cultural competencies OR cultural competence OR cultural sensitivity OR cultural competencies OR cultural competence OR cultural sensitivity OR culture OR language barriers) | 94,070 |
| **2** | (Indigenous OR ethnic minority OR ethnic minorities OR racial minorities OR racial minority OR ethnic minority OR ethnic minorities OR racial minorities OR racial minority) | 19,646 |
| **3** | (Europe OR Russia OR Germany OR United Kingdom OR France OR Italy OR Spain OR Ukraine OR Poland OR Romania OR Netherlands OR Belgium OR Czech Republic OR Czechia OR Greece OR Portugal OR Sweden OR Hungary OR Belarus OR Austria OR Serbia OR Switzerland OR Bulgaria OR Denmark OR Finland OR Slovakia OR Norway OR Ireland OR Croatia OR Moldova OR Bosnia and Herzegovina OR Albania OR Lithuania OR Macedonia OR Slovenia OR Latvia OR Estonia OR Montenegro OR Luxembourg OR Malta OR Iceland OR Andorra OR Monaco OR Liechtenstein OR San Marino OR Holy See OR Andalusia OR Galicia OR Asturias OR Basque OR Aragon OR Catalonia OR Brittany OR Normandy OR Occitania OR Guernsey OR Jersey OR Cornwall OR Wales OR Man OR Scotland OR Flanders OR Wallonia OR Lorraine OR Alsace OR Romansch OR Savoy OR Aosta OR Piedmont OR Liguria OR Corsica OR Sardinia OR Bavaria OR Friesland OR Faroe OR Samiland OR Sami OR Swedes OR Aland Islands OR Livonia OR Voro OR Scania OR Danes OR Kashubia OR Sorbia OR Silesia OR Moravia OR Transdniestria OR Szekely land OR Napolitania OR Istria OR Lombardy OR Roma) | 386,112 |
| **4** | women OR gender | 545,679 |
|  | **#1 AND #2 AND #3 AND #4** | **89** |
